# Conducting Salts Govern Thermal Boundary Conductance across Solid Electrode/Organic Liquid Electrolyte Interfaces in Lithium-Ion Batteries

**DOI:** 10.1021/acsnano.5c13221

**Published:** 2025-12-02

**Authors:** C. Jaymes Dionne, Patrick E. Hopkins, Arijit Bose, Ashutosh Giri

**Affiliations:** † Department of Mechanical, Industrial and Systems Engineering, 4260 University of Rhode Island, Kingston, Rhode Island 02881, United States; ‡ Department of Mechanical and Aerospace Engineering, 2358 University of Virginia, Charlottesville, Virginia 22904, United States; § Department of Materials Science and Engineering, 2358 University of Virginia, Charlottesville, Virginia 22904, United States; ∥ Department of Physics, 2358 University of Virginia, Charlottesville, Virginia 22904, United States; ⊥ Department of Chemical Engineering, 4260 University of Rhode Island, Kingston, Rhode Island 02881, United States

**Keywords:** thermal boundary conductance, lithium-ion batteries, polymer electrolyte, nanoscale thermal transport, solid−liquid interfaces

## Abstract

Thermal boundary resistance at material interfaces poses a major challenge to effective heat dissipation in lithium-ion batteries, particularly at the interface between solid electrodes and organic liquid-based electrolytes. Despite its critical role in thermal management, the nanoscale mechanisms governing interfacial heat transfer in these systems remain poorly understood. Here, we employ all-atom molecular dynamics simulations to investigate heat transport across the interface between lithium cobalt oxide (LCO) electrodes and a liquid electrolyte mixture of ethylene carbonate and ethyl methyl carbonate (3:7 mass ratio) containing either LiPF_6_ or LiTFSI salts at concentrations ranging from 0.05 to 2 M. Our results show that thermal boundary conductance is highly sensitive to both the identity of the conducting salt and the degree of lithium-ion adsorption on the LCO surface. While thermal boundary conductance can be as low as 20 MW m^–2^ K^–1^ at room temperature—comparable to the resistance of a ∼2 μm silicon layer—increased lithium surface coverage enhances vibrational coupling and significantly increases thermal boundary conductance. We also find that larger anions such as TFSI^–^ enable better interfacial heat transfer than smaller PF_6_^–^ anions, which disrupt vibrational bridging at high lithium densities. Spectral analyses reveal that adsorbed lithium ions facilitate low-frequency vibrational coupling, especially in the LiTFSI system where the contributions from the transverse phonon modes in the solid are crucial. These findings underscore the critical role of salt-specific interfacial structuring and vibrational dynamics in modulating heat transfer, offering key design insights for thermally optimized, high-performance lithium-ion batteries.

## Graphical abstract

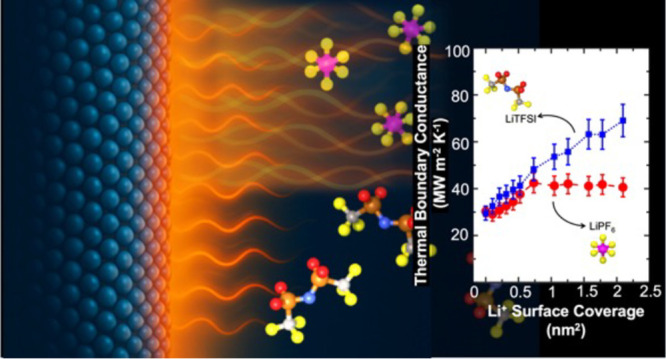

## Introduction

Thermal boundary resistance at material interfaces is a well-known obstacle to effective heat transfer in many technologies.^1–3^ This issue is particularly significant in lithium-ion batteries (LIBs), where heat transfer is hindered by the high density of interfaces between solid electrodes and organic liquid-based electrolytes.^4–6^ Despite its importance, the nanoscale mechanisms of heat transport across these solid electrode/organic liquid electrolyte interfaces remain poorly understood, limiting our ability to optimize the overall thermal performance of rechargeable batteries.

Effective thermal management of LIBs is essential in battery system design to ensure efficient heat dissipation during charge and discharge cycles. As LIBs become more widely used in electric vehicles and large-scale energy storage systems, there is a growing emphasis on enhancing their energy density and charge/discharge rates.^7–11^ However, these advancements lead to increased overheating during operation. Consequently, a thorough understanding of the thermal behavior of battery components is crucial to ensure the safe functioning of high-energy, high-rate LIBs.

Thermal transport in LIB components has been explored using multiscale models that account for heat generated from ohmic losses due to ionic and electronic resistance, kinetic effects from electrochemical reactions in the electrodes, and entropic changes from intercalation processes in active materials.^12,13^ Despite their utility, these models rely on macroscopic properties that overlook the intricate nature of heat transfer at the nanoscale.^14–16^ Moreover, many of these models aim to describe thermal behavior at the cell level without fully understanding the nanoscale factors that govern thermal transport. In this context, atomistic simulations offer valuable insights into nanoscale heat transfer mechanisms within battery materials that are often excluded from multiscale approaches. For example, recent molecular dynamics (MD) simulations have shed light on Li-ion transport in electrolytes,^17^ energy barriers at active material/electrolyte interfaces,^18–20^ and the formation of the solid electrolyte interphase.^21–23^ Nevertheless, there remains a scarcity of atomistic studies specifically focused on heat transfer within battery components, particularly interfacial thermal transport at the active material/electrolyte boundary on the nanoscale.

Although nanoscale heat transfer in Li-ion batteries has received limited attention, several studies have revealed key bottlenecks that constrain efficient thermal transport—most notably, the dynamic evolution of thermal properties during cycling and the dominant role of interfacial thermal resistance.^24–29^ Both experimental and computational investigations have shown that the thermal conductivity of lithium cobalt oxide is electrochemically tunable: delithiation can reduce it by up to ∼32%, accompanied by a drop in elastic modulus from ∼325 to ∼225 GPa.^24,25^ These changes indicate that phonon transport is highly sensitive to lithium content, with the reduction in thermal conductivity largely attributed to increased phonon scattering from lithium vacancies and disorder-induced nonpropagating vibrational modes. Such findings make clear that the thermal conductivity of electrode materials is not static but varies with cycling. Building on this, Song et al.^29^ highlight the direct link between nanoscale thermal transport limitations and battery safety concerns. Their review emphasizes that nonuniform heat generation, localized hotspots, and insufficient heat dissipation can accelerate the onset of thermal runaway. Importantly, they identify interfacial thermal resistance, between electrodes, separators, and current collectors, as a critical bottleneck, underscoring that interfacial engineering is as crucial as improving bulk conductivity. Consistently, Zeng et al.^28^ and Alosious et al.^27^ report interfacial thermal resistance values of <40 MW m^–2^ K^–1^ for carbon-based electrodes, comparable to those observed in this work at the LCO/polymer electrolyte interface without lithium adsorption. This demonstrates that interfacial heat transport is a pervasive challenge across electrode/electrolyte systems.

Within a battery electrode (cathode), active material particles form a porous structure bound together by a polymer binder that incorporates carbon additives to improve electronic conductivity throughout the electrode. The liquid electrolyte infiltrates this porous network, facilitating lithium-ion transport across the electrode. Due to the high density of active material/electrolyte interfaces in this structure, interfacial thermal resistance can play a critical role in limiting heat transfer. Furthermore, the accumulation of lithium ions at the surfaces of active material particles, which governs the kinetics of the charging process,^30^ may also impact interfacial heat transfer. However, this effect remains largely unexplored.

In general, while heat transfer across solid/solid interfaces has been widely studied,^1,2,31^ solid/liquid interfaces have received less attention, largely due to the experimental challenges associated with measuring thermal properties at such interfaces.^32^ In this regard, MD simulations offer a powerful approach for probing the nanoscale behavior of interfacial heat transfer at solid–liquid boundaries. Computational studies have revealed the influence of factors such as wetting properties and adhesion energy, surface roughness, and the structuring of liquid molecules near the interface on the solid/liquid thermal boundary conductance (TBC; *h*_K_ = 1/*R*_K_, where *R*_K_ is the thermal boundary resistance).^33–42^ However, most of these investigations have focused on simplified systems using Lennard-Jones (LJ) fluids or single-component liquids. As a result, the heat transfer mechanisms identified may not directly translate to realistic battery electrolytes, where the wide variety of organic solvent blends and conducting salt additives used in LIBs often as complex ternary or quaternary mixtures, further complicates accurate predictions of interfacial thermal transport.

In this work, we utilize all-atom molecular dynamics simulations to explore the underlying mechanisms of heat transfer at the interface between solid cathode electrodes and organic liquid-based electrolytes, with relevance to lithium-ion battery applications. Our investigation centers on a widely used interface composed of lithium cobalt oxide (LCO) as the electrode material and a liquid electrolyte blend of ethylene carbonate (EC) and ethyl methyl carbonate (EMC) in a 3:7 mass ratio. The electrolyte includes varying concentrations (0.05–2 M) of either lithium hexafluorophosphate (LiPF_6_) or lithium bis­(trifluoromethanesulfonimide) (LiTFSI) as the conducting salt. We find that the efficiency of interfacial heat transfer is strongly affected by both the choice of salt and the extent of lithium ion adsorption on the electrode surface. Our simulations reveal that the thermal boundary conductance at the LCO/electrolyte interface can be as low as 20 MW m^–2^ K^–1^ at room temperature—comparable to the thermal resistance of a ∼2-μm-thick silicon layer. Importantly, increasing lithium ion adsorption at the interface can more than double the TBC, underscoring the critical role of interfacial structuring. Unlike traditional perspectives that emphasize the liquid’s density depletion layer as the dominant factor in heat transfer, our results demonstrate that ion size and anion–cation interactions near the surface are equally, if not more, influential—especially under high lithium coverage. Our vibrational density of states and spectrally resolved heat flux calculations further reveal that adsorbed lithium ions act as vibrational bridges, enhancing low-frequency coupling between the solid and liquid phases—particularly in the LiTFSI system, where transverse modes and vibrations below 10 THz dominate interfacial heat transport. Conversely, smaller anions like PF_6_^–^ can approach the surface more closely and, in doing so, interfere with vibrational coupling between the LCO and the electrolyte, leading to diminished TBC. Overall, our atomistic insights highlight the importance of ion-specific effects in governing interfacial heat transfer and suggest that careful selection of electrolyte composition—particularly the conducting salt—can be a powerful strategy for enhancing thermal management in next-generation lithium-ion batteries.

## Results and Discussion

We use the Large-scale Atomic/Molecular Massively Parallel Simulator (LAMMPS) package^43^ to perform our simulations on LCO/organic liquid electrolyte interfaces with 0.0.5–2 M LiPF_6_ or LiTFSI salt. The two different salts were chosen as they are commonly used in liquid electrolytes and have a noticeable difference in size (94 Å^3^ vs 179 Å^3^ for PF_6_^–^ and TFSI^–^, respectively). Typical concentrations of salts in liquid electrolytes vary by applications and are in the range chosen for this work, but typically 1 M concentrations are used for practical applications since higher concentrations lead to higher viscosity and lower conductivity.^44^

We begin by calculating the thermal boundary conductances (TBCs) between the solid electrode and liquid electrolytes, examining how they change as the salt concentration varies from 0 to 2 M, initially assuming no adsorbed lithium ions on the LCO surface. Figure 1a presents the TBC results as a function of salt concentration in the liquid electrolyte. For the LiPF_6_ system, the TBC remains relatively constant across concentrations, with any changes falling within the uncertainty range. However, for the LiTFSI system, the TBC drops by approximately 30% at higher concentrations. We attribute this difference to the way the liquid electrolyte organizes itself near the LCO surface, which differs significantly between the smaller PF_6_^–^ anion and the larger TFSI^–^ anion.

**Figure 1:**
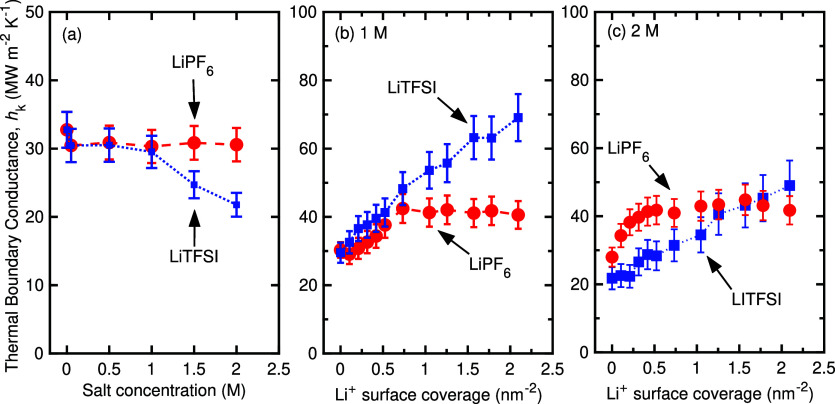
(a) Thermal boundary conductance (TBC) as a function of conducting salt concentration for the LiPF_6_- and LiTFSI-based systems. For both systems, at low concentrations (≤1 M), the TBC approaches the TBC when no salt is present in the liquid electrolyte. For higher concentrations, the TBC of the LiTFSI system reduces by ∼30*%,* while the LiPF_6_ remains unaffected. Thermal boundary conductance as a function of the surface coverage of Li^+^ at the LCO/electrolyte interface for the two different systems at (b) 1 M and (c) 2 M salt concentrations.

Before delving into the structural effects of the electrolyte, we next analyze how different surface coverages of adsorbed lithium ions affect the TBCs at 1 and 2 M concentrations for both salt systems studied in this work. Figure 1b,c display how the thermal boundary conductance varies with the coverage of adsorbed lithium ions on the LCO surface at 1 and 2 M salt concentrations, respectively. Note, the lithium-free control case, represented by the 0 nm^–2^ Li coverage data in Figure 1 serve as baseline systems without any Li species at the interface. By systematically comparing these Li-free domains with those containing LiTFSI and LiPF_6_, we observe a clear and consistent enhancement in TBC upon Li adsorption.

In the 1 M case (Figure 1b), TBC values for both LiPF_6_ and LiTFSI systems are comparable at low Li-ion surface coverages (below ∼0.75 nm^–2^). However, as lithium coverage increases beyond this threshold, the behavior diverges: for LiPF_6_, the TBC saturates, whereas for LiTFSI, the TBC continues to increase steadily. This difference results in a 2-fold variation in TBC between the two salts at high lithium coverages, highlighting the significant influence of salt identity on interfacial heat transfer behavior.

For the 2 M salt concentration, as previously noted, TBCs are generally lower for the LiTFSI system compared to the LiPF_6_ system. Nonetheless, similar to the 1 M case, increasing lithium ion adsorption leads to a modest and a monotonic increase in TBC for LiTFSI with increasing lithium-ion surface coverage. The remainder of this study will explore the underlying mechanisms that drive these distinct TBC trends for different salts, offering a detailed understanding of how salt-specific interactions affect interfacial thermal transport.

As previous studies have shown that the structuring of liquid near solid interfaces can strongly impact heat transfer, we begin by comparing the anion number density within 5 Å of the LCO/electrolyte interface for LiPF_6_ and LiTFSI systems. This comparison focuses on the 1 M concentration at the maximum lithium ion adsorption on the LCO surface, where the largest variation in TBC is observed (see Figure 2). In the case of LiPF_6_, the PF_6_^–^ anion density near the interface is higher and increases with greater Li^+^ adsorption, due to the anion’s smaller size and higher mobility. Prior research has established that the size of anions and solvent molecules significantly affects Li^+^ transport, especially near electrode interfaces.^17,18^ Since PF_6_^–^ is nearly half the size of TFSI^–^ (94 Å^3^ vs 179 Å^3^),^45^ it more readily accumulates at the interface when lithium coverage is high. This increased accumulation of PF_6_^–^ correlates with the point where TBC deviates from its previously linear trend, occurring around Li^+^ surface coverages above ∼0.75 nm^–2^, indicating that smaller anions like PF_6_^–^ play a key role in limiting heat transfer at these higher coverages. By contrast, in the LiTFSI system, the increase in TBC follows a trend similar to that observed in a hypothetical salt-free electrolyte (see Figure S3), suggesting that TFSI^–^ anions, being larger and less mobile, have a weaker impact on interfacial heat transfer in this regime.

**Figure 2:**
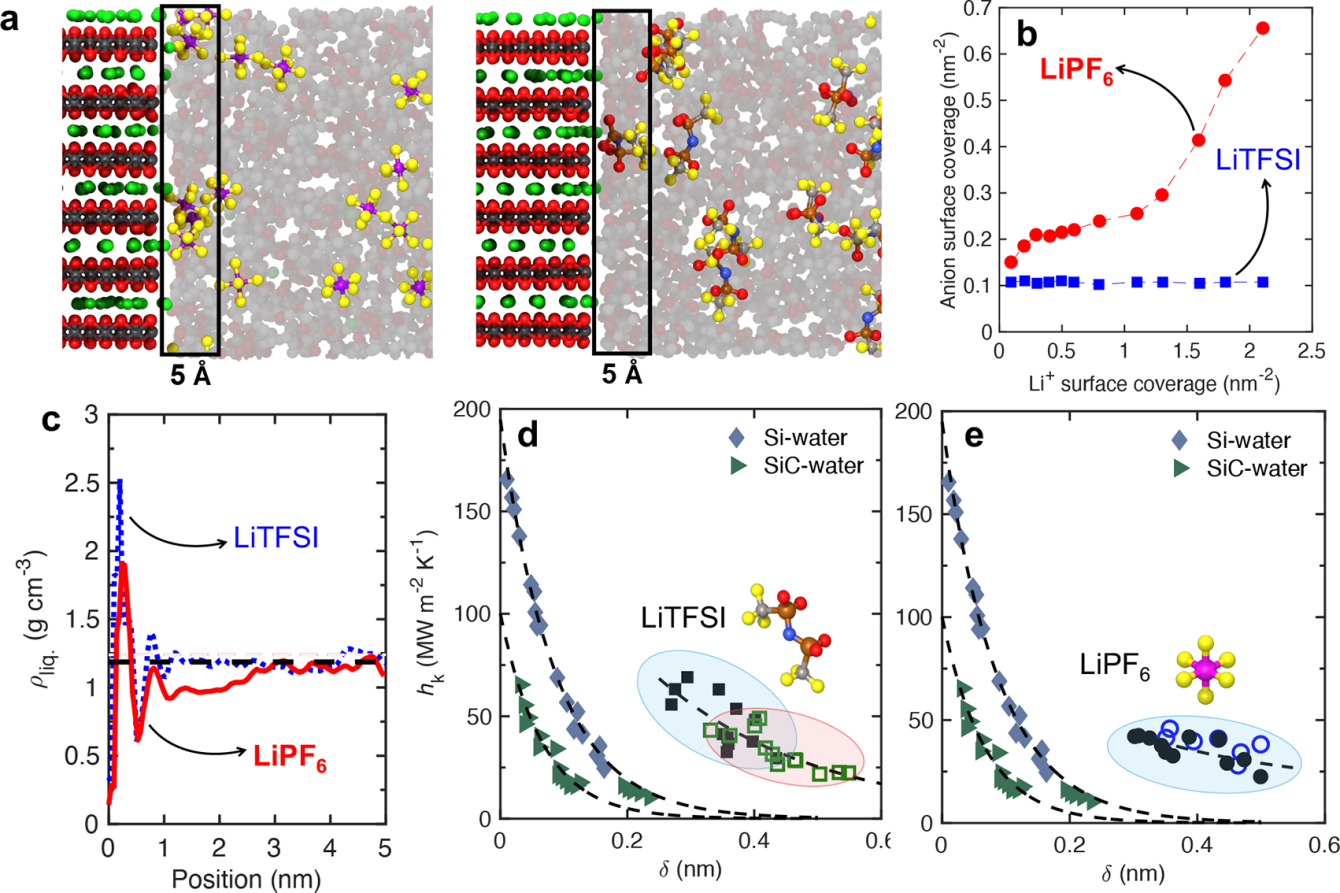
(a) Schematic illustrations show how anion salts accumulate at the LCO/electrolyte interface under conditions of high lithium ion surface coverage (∼2 nm^–2^) for the PF_6_^–^ (left panel) and TFSI^–^ (right panel) systems. Because PF_6_^–^ is smaller in size, it can more easily navigate through the EC/EMC solvent environment and reach the interface. In contrast, TFSI^–^, which is approximately twice the size of PF_6_^–^, is sterically hindered from approaching the LCO surface. (b) The number density of anions within 5 Å of the interface is plotted as a function of lithium ion surface coverage. As Li^–^ coverage increases, significantly more PF_6_^–^ anions are drawn toward the interface due to strong Coulombic attraction. However, the larger TFSI^–^ ions remain excluded from the interfacial region regardless of Li^+^ density due to their bulkiness. (c) Local liquid density profiles as a function of distance from the LCO surface for both salt systems at 1 M salt concentration and highest lithium surface coverage condition. Finally, thermal boundary conductance is plotted against the density depletion length for (d) LiTFSI and (e) LiPF_6_ systems, with comparisons to previously reported Si/water and SiC/water interfaces from refs 46 and 38 , respectively. Note, the uncertainties in our values of *h*_K_ are ∼15–20%.

When considering the TBC of solid–liquid interfaces, the structuring of the liquid at the interface is often considered as a strong contributor to the thermal transport across the interface. In this regard, the density depletion length, δ, is often used to describe the interfacial liquid structuring and the effective contact between the solid and the liquid atoms.^46^ The density depletion length is given by,

$$\delta =\int_{0}^{\infty }\left(1-\frac{\rho_{S}(z)}{\rho_{S}^{b}}-\frac{\rho_{L}(z)}{\rho_{L}^{b}}\right)dz$$ 1

where δ is the depletion length, ρ is the mass density, *S* and *L* denote the solid and liquid, respectively, and *b* denotes the value of the bulk property. In essence, the depletion length characterizes how the liquid density decreases near the solid–liquid interface as a result of interfacial interactions. As illustrated in Figure 2c, the electrolyte density profile near the LCO surface differs significantly between the two salts. In the case of LiPF_6_, the liquid density near the interface remains distinct from the bulk value even at distances up to 3 nm away from the LCO surface. This extended deviation from bulk behavior is captured by the concept of the density depletion length, which we will examine in more detail in the following discussion.

Figure 2d,e present our calculated thermal boundary conductance values as a function of density depletion length for the LiTFSI and LiPF_6_-based electrolyte systems, respectively. For comparison, we also include TBC predictions from MD simulations for Si/water and SiC/water interfaces from previous studies (refs 46 and 38 ). Note, we chose to compare our results to the water-based system because most of the prior works focusing on TBC across solid/liquid interfaces have considered generic Lennard-Jones-based ‘toy models’ and therefore, these values present realistic interfacial conductances with a polar liquid (as is the case for our electrolyte systems). Prior research suggests that the depletion length effectively captures variations in TBC at solid/liquid interfaces, typically exhibiting an exponential decrease in TBC with increasing depletion length—as seen in the Si and SiC systems. In our electrolyte systems, we observe a general trend of decreasing TBC with increasing depletion length. However, this relationship is not as strongly correlated as the exponential decay reported for Si- or SiC-based interfaces.

Among the two electrolytes, the LiTFSI system shows a clearer correlation: the depletion length increases at 2 M salt concentration, where the TBCs are lower than those at 1 M. In contrast, the LiPF_6_ system shows minimal changes in both depletion length and TBC values with increasing salt concentration, indicating a weaker dependency on this structural parameter.

The density depletion length alone cannot fully account for the observed differences in thermal boundary conductance between the two electrolyte systems. This is evident in Figure 3a, where TBCs differ by approximately 75% even at the same depletion length of around 0.3 nm. Despite a weaker correlation, the density depletion length still provides reasonable estimates of the approximate TBC in our complex electrolyte systems. However, to uncover the additional underlying mechanisms influencing TBC at the solid electrode/organic liquid electrolyte interfaces, we analyze the vibrational density of states (DOS) for the solid LCO electrode, the electrolyte, and the interfacial lithium ions adsorbed on the electrode surface.

**Figure 3:**
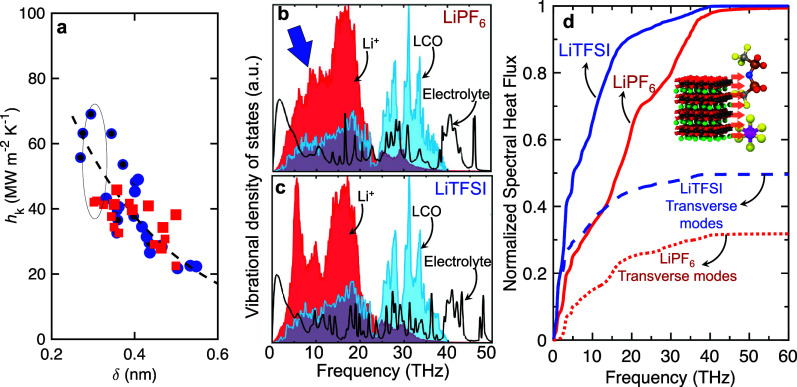
(a) Thermal boundary conductances for the LiTFSI-based (blue circles) and the LiPF_6_-based (red squares) systems as a function of the density depletion length, highlighting that even for the same depletion length, the thermal boundary conductances can vary drastically for the two systems. The uncertainties in our values of *h*_K_ are ∼15–20%. (b) Vibrational DOS of the interfacial Li^+^, LCO, and electrolyte for the (b) LiPF_6_ and the (c) LiTFSI systems with 2.0 nm^–2^ lithium-ion surface coverage at the interface. (d) Spectral contributions to the TBC between solid (LCO) and PF_6_^–^ (black) or TFSI^–^ (red). For the TFSI^–^ system, frequencies below ∼20 THz dominate the heat transfer between LCO and TFSI^–^ with better transverse mode coupling across the interface.

Figure 3b,c display the DOS profiles for both LiPF_6_ and LiTFSI systems at the same depletion length (∼0.3 nm), where the significant TBC variation is observed. The DOS of the liquid electrolytes shows a distinct low-frequency peak, typical of liquids,^47^ which represents the vibrational modes that primarily carry heat in the fluid.^48^ The adsorbed lithium ions exhibit vibrational modes that align more closely with these low frequencies than the broader-spectrum DOS of the LCO solid, enabling better vibrational coupling. This frequency overlap explains the observed increase in TBC at low Li^+^ surface coverage levels (below ∼0.75 nm^–2^), as the lithium ions act as a ‘vibrational bridge’—facilitating energy transfer between the high-frequency modes of the solid LCO and the low-frequency modes of the liquid. This bridging mechanism is similar to phenomena reported at solid/solid interfaces, where inserting a thin interlayer with an intermediate DOS enhances TBC by providing matching vibrational modes between two dissimilar materials.^49–52^ Although such interfacial layers introduce additional boundaries, the improved vibrational coupling they provide can lead to greater overall interfacial heat transfer.

At the highest levels of lithium ion surface coverage, it is noteworthy that although the vibrational spectra of the two electrolytes are quite similar, the vibrational density of states of the adsorbed lithium ions differ between the LiPF_6_ and LiTFSI systems. Specifically, in the LiPF_6_ system (Figure 3b), the low-frequency (∼10 THz) peak in the DOS of the adsorbed lithium ions is significantly weaker compared to the more pronounced peak observed in the LiTFSI system (Figure 3c). This distinction in vibrational behavior offers insight into the ∼75% difference in TBC observed between the two systems at high lithium coverage. The stronger low-frequency peak (∼10 THz) in the LiTFSI case aligns more effectively with the dominant low-frequency modes of the liquid electrolyte, enabling more efficient energy transfer. This enhanced overlap improves the role of the adsorbed lithium ions as a ‘vibrational bridge’ between the high-frequency modes of the LCO electrode and the low-frequency modes of the electrolyte, thereby increasing the thermal boundary conductance.

To further substantiate the ‘vibrational bridge’ mechanism, we performed simulations in which the vibrational spectrum of the LCO domain was artificially modified by fictitiously increasing or decreasing its atomic mass, thereby producing either stronger or weaker overlap with the 20 THz vibrational modes of the electrolyte. The extent of this vibrational overlap was quantified from the vibrational density of states obtained via power spectral density analysis, enabling calculation of the percentage of overlapping area (Figure S13). Relative to the control case of a bare LCO surface in contact with the liquid electrolyte, lithium ion adsorption enhances this vibrational overlap, effectively serving as a ‘bridge’ for the heat-carrying (<20 THz) modes across the LCO/electrolyte interface (Figure S14). Nonetheless, it is important to note that vibrational mode overlap alone does not fully determine interfacial heat transfer; liquid structuring and depletion length effects also play significant roles. As a result, even at comparable overlap percentages, the bare LCO surface and the LCO surface with a lithium ion coverage of 2 nm^–2^ exhibit substantially different thermal boundary conductances (Figure S14).

To support the idea that low-frequency vibrational modes of both the adsorbed lithium ions and the LCO electrode—which align with the low-frequency peak of the electrolyte—are key contributors to heat transfer across the LCO/organic liquid electrolyte interface, we conducted spectrally resolved heat flux calculations. These results, shown in Figure 3d, correspond to conditions where both electrolyte systems have similar density depletion lengths but differ significantly in TBC values (∼75%).

In the LiTFSI system, vibrational modes below 10 THz account for up to 70% of the total heat flux across the interface. In contrast, in the LiPF_6_ system, the contribution from these low-frequency modes is notably reduced, with higher-frequency modes playing a more dominant role in thermal transport. These findings indicate that in the LiTFSI system, the adsorbed lithium ions enable stronger vibrational coupling between the LCO surface and the liquid electrolyte, particularly through low-frequency vibrations. This enhanced coupling is less effective in the LiPF_6_ system, where the smaller anion appears to result in weaker interaction between the solid and liquid phases.

Interestingly, the transverse vibrational modes contribute more significantly to heat transfer at the lithium-adsorbed LCO interface in the LiTFSI system—accounting for approximately 50% of the total heat flux—compared to only about 30% in the LiPF_6_ system. This trend aligns with previous studies on general solid/liquid interfaces, which suggest that stronger interfacial bonding enhances the coupling of transverse phonons and leads to higher thermal boundary conductance.^1,40,53^ Additionally, Ramos-Alvarado et al.,^46^ observed that in Si/water systems, a reduced density depletion length (which correlates with higher TBC) and the presence of water entrainment contribute to increased transverse mode-mediated heat transfer. These findings support our spectral analysis, which shows lower transverse mode contributions in the LiPF_6_ system relative to LiTFSI. However, unlike conventional solid/liquid systems—such as Si/water or Lennard-Jones model systems—TBC in solid electrode/organic liquid electrolyte interfaces may vary substantially even when the density depletion length is similar. This is primarily due to the distinct vibrational behavior of adsorbed lithium ions, which is strongly influenced by the type of conducting salt in the electrolyte. It should be noted that the applied harmonic restraint acts only along the direction perpendicular to the interface, primarily influencing the longitudinal modes. However, supplementary simulations confirm that this restraint does not modify their heat-transport characteristics. The lithium ions, on the other hand, were free to move parallel to the interface, corresponding to the transverse modes. Since the transverse modes of the adsorbed lithium ions remain unaffected, the observed trends in the calculated thermal boundary conductance are not significantly influenced by the applied harmonic restraint (Figure S5).

In this work, we assume that the adsorbed lithium ions have already lost their solvation shells, and we do not account for the effects of dynamic screening. While our simulations include electrostatic interactions, they do not explicitly capture the solvation structure or time-dependent screening effects that become significant during rapid charge/discharge processes. Such dynamic phenomena could influence variations in the thermal boundary conductance, but their investigation lies beyond the scope of the present study and warrants future exploration. It is also important to note here that, while we do not include them in our simulations, real-world electrodes form a solid-electrolyte interphase (SEI) layer on the surface of the electrode during cycling. This additional layer could act as an additional vibrational bridging mechanism that enhances the interfacial heat transfer between the electrode and liquid electrolyte. However, given the complexity of accurately modeling the SEI layer and our present focus on the effects of electrolyte salt and lithium ion adsorption, a detailed investigation of the SEI layer’s role is left for future work.

In addition to exploring the fundamental mechanisms governing thermal transport across solid and organic liquid electrolyte interfaces, this study highlights a critical design insight for future lithium-ion batteries: the need to minimize interfacial thermal resistance, which can be comparable to the resistance of a 25 nm thick electrolyte layer with a thermal conductivity of ∼0.5 W m^–1^ K^–1^.^14,54^ To evaluate how the electrode/electrolyte interface affects overall heat transfer through porous electrodes, we employ a thermal resistor model, incorporating the Kapitza lengths for the electrode, electrolyte, and their interface. For our calculations, we use the bulk thermal conductivities of the electrode and electrolyte materials, and apply both high and low Kapitza resistance values measured for LiPF_6_ and LiTFSI electrolytes at 1 M concentration—a standard for most commercial Li-ion batteries. Given that electrode pore sizes vary widely, from micropores (∼10 nm) to macropores (>1 μm),^55,56^ we estimate the total thermal resistance based on an effective electrolyte thickness corresponding to these pore sizes. Our analysis shows that the thermal resistance contributed by the active material/electrolyte interface can range from as low as 0.4% (where the low thermal conductivity of the electrolyte dominates) to as high as 60% of the total electrode thermal resistance—depending strongly on the pore size distribution (Figure 4). These findings underscore the importance of accounting for interfacial thermal resistances in porous electrode design, especially since excessive heat generation at high charge/discharge rates can result in performance degradation or even thermal runaway.

**Figure 4:**
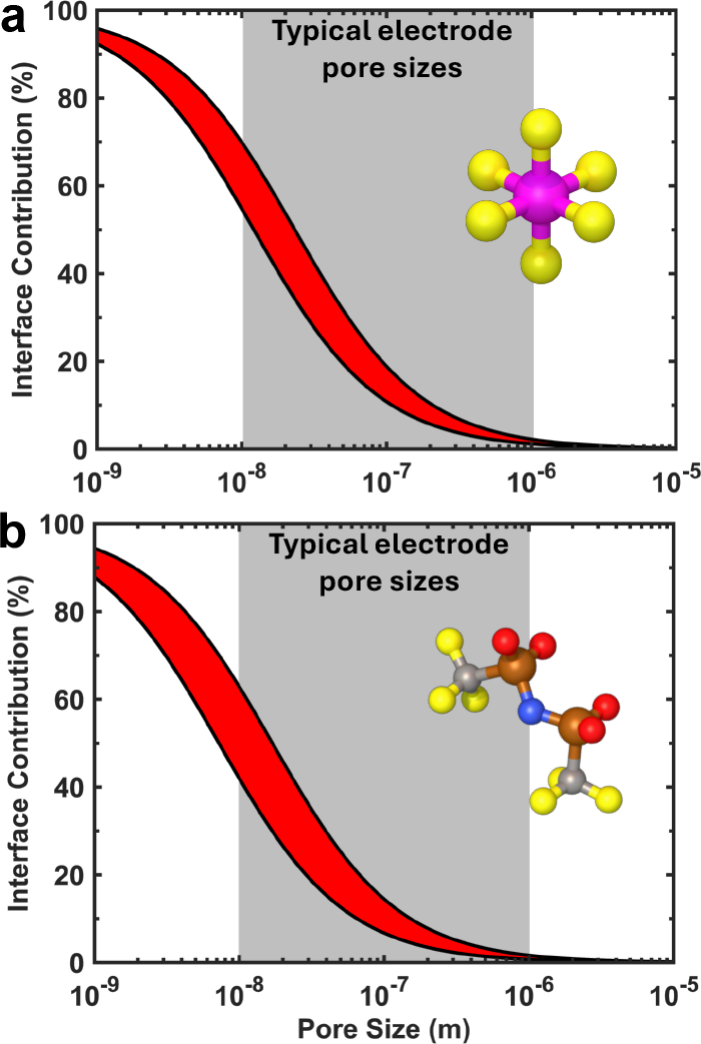
Thermal resistance contributed by the active material/electrolyte interface to the total thermal resistance in the (a) LiPF_6_ and (b) LiTFSI systems. Depending on the choice of salt and average pore size, the interfacial thermal resistance can reach up to 60*%* for typical electrodes today, but will be even more significant as the pore size decreases further (as is the case for nanofabricated active material particles).

In recent years, there has been growing interest in using nanostructured active material particles with dimensions of 100 nm or less.^57–60^ While typical commercial lithium based particles use micron-sized active particles, nanoscale active particles are being explored currently because they have increased surface area allowing faster charge/discharge, full capacity utilization and reduced intraparticle differential stresses during charging and discharging which reduces particle pulverization.^61^ However, these smaller particles also reduce electrode pore sizes to under 10 nm (based on random close packing of ∼17 nm diameter particles). When combined with high cycling rates, this results in greater heat generation within the electrode. Under such conditions, the thermal resistance at the nanoparticle–electrolyte interface can dominate the total thermal resistance of the electrode, regardless of the type of electrolyte salt used. Consequently, this interfacial resistance must be thoroughly accounted for in thermal modeling and in the design and manufacturing of electrodes incorporating nanoparticles.

## Conclusions

In this work, we investigated the intrinsic mechanisms dictating thermal boundary conductance at the interface between a lithium cobalt oxide (LCO) electrode and liquid electrolytes containing LiPF_6_ and LiTFSI salts. Our results reveal that interfacial heat transfer is strongly influenced by both the identity of the conducting salt and the extent of lithium ion adsorption on the electrode surface. While TBC remains relatively constant for LiPF_6_ across salt concentrations, it decreases significantly for LiTFSI at higher concentrations—underscoring the role of interfacial structuring and anion-specific interactions in thermal transport. Through structural and spectral analyses, we demonstrate that smaller and more mobile PF_6_^–^ anions accumulate more readily near the LCO surface at high lithium coverages, limiting vibrational coupling and thereby suppressing TBC. In contrast, the larger TFSI^–^ anions lead to weaker interfacial structuring and enable a more continuous increase in TBC with lithium adsorption. Our vibrational density of states and spectrally resolved heat flux calculations further reveal that adsorbed lithium ions act as vibrational bridges, enhancing low-frequency coupling between the solid and liquid phases—particularly in the LiTFSI system, where transverse modes and vibrations below 10 THz dominate interfacial heat transport. Importantly, we find that density depletion length alone cannot fully explain the observed TBC trends, especially given the ∼75% difference in TBC between the two salt systems at similar depletion lengths. Instead, our findings point to a salt-specific modulation of interfacial vibrational coupling, driven by differences in ion size and the resulting interfacial liquid structuring. Finally, we show that these interfacial thermal resistances can contribute significantly to the overall thermal resistance of porous electrodes, particularly in architectures featuring nanoporous structures or small particle sizes. In such systems, interfacial resistance may account for up to 60% of total thermal resistance, highlighting its critical role in thermal management for next-generation lithium-ion batteries. These insights emphasize the need to incorporate accurate interfacial thermal resistance models into battery design, especially as electrode structures become increasingly nanostructured and energy-dense.

## Methods

We use the Large-scale Atomic/Molecular Massively Parallel Simulator (LAMMPS) package^43^ to perform our simulations on LCO/organic liquid electrolyte interfaces with 0.0.5–2 M LiPF_6_ or LiTFSI salt. The two different salts were chosen as they are commonly used in liquid electrolytes and have a noticeable difference in size (94 Å^3^ vs 179 Å^3^ for PF_6_^–^ and TFSI^–^, respectively). Typical concentrations of salts in liquid electrolytes vary by applications and are in the range chosen for this work, but typically 1 M concentrations are used for practical applications since higher concentrations lead to higher viscosity and lower conductivity.^44^

Consistent with prior works on similar systems,^17,18,62,63^ the universal force field (UFF)^64^ was used to model the LCO structure and the OPLS all-atom force field^63^ was used to model the liquid electrolyte molecules. All our force field parameters are given in the Supporting Information. While the UFF potential is a generalized force field and not specifically parametrized for transition metal oxides, our focus here is to assess how the choice of electrolyte salt and the degree of lithium-ion adsorption at the surface affect the qualitative trends in thermal boundary conductance at the LCO/liquid electrolyte interface. Since the same LCO structure is employed across both electrolyte systems, any observed variations can be attributed directly to differences in salt chemistry and lithium-ion adsorption. However, to get accurate predictions of the LCO/liquid electrolyte interfacial heat transfer, one would require more accurate potentials (such as those based on ab initio trained machine learning potentials for the LCO domain, which is beyond the scope of the current work but deserves further work). Therefore, we refer to our simulated cathode structures as a “model LCO domain” to emphasize that it serves as a representative framework for exploring fundamental interfacial mechanisms rather than reproducing the exact thermal conductivity of crystalline LCO.

The PACKMOL package^65^ was used to generate a random configuration of the electrolyte molecules. The cross-section of the LCO electrodes are chosen to be ∼30 × 30 Å^2^ with a thickness of 20 Å and the liquid electrolyte domain in contact with the LCO has a length of ∼80 Å in length; note, as size effects can influence the interfacial heat transfer processes,^1^ our choice of the size of our domains has negligible effect on the reported thermal boundary conductances. An example schematic of our simulation domain, with the profile of the temperature gradient induced across the domain, along with the different types of molecules used in the liquid electrolyte are shown in Figure 5.

**Figure 5:**
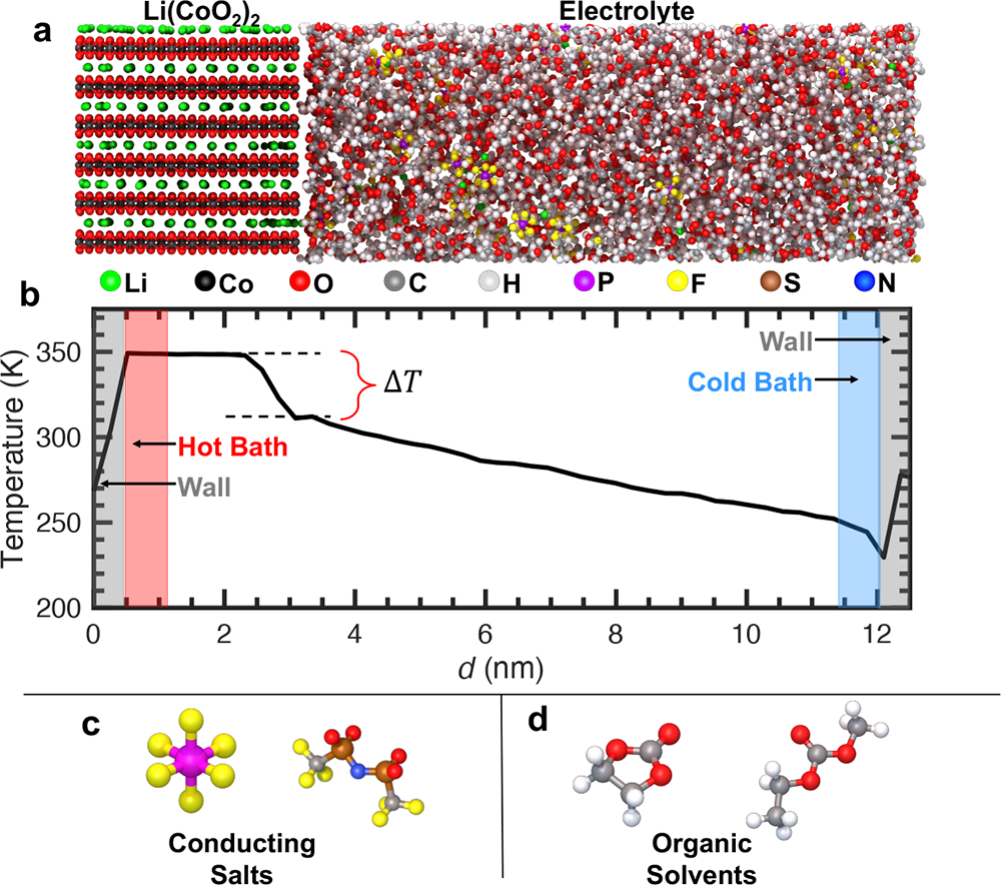
(a) Schematic representation of our simulation domain. A delithiated LCO electrode (Li­(CoO_2_)_2_) is on the left, in contact with a 3:7 wt % EC/EMC electrolyte with 1 M LiPF_6_ conducting salt. The electrode thickness is ∼28 Å, while the electrolyte is ∼80 Å. (b) Applying a hot and a cold bath at the ends of the simulation domain, we create a steady state temperature profile, from which we predict the thermal boundary conductance by considering the temperature drop at the solid electrode/polymer electrolyte interface. We investigate the role of two different conducting salt additives, (c) LiPF_6_ and LiTFSI, on the thermal transport across the LCO/electrolyte interface. The electrolyte consists of a mixture of (d) ethylene carbonate (EC) and ethylmethyl carbonate (EMC) molecules.

After the structures were generated, energy minimization scheme was implemented and the system temperature was specified to 300 K under the NPT ensemble (constant number of atoms, pressure, and temperature held constant) for a total of 2 ns. Note, at higher temperatures, electrolytes in lithium-ion batteries may undergo decomposition and chemical reactions that classical nonreactive force fields cannot capture. It is important to note that at elevated temperatures, electrolytes in lithium-ion batteries may undergo decomposition and chemical reactions that cannot be described by classical nonreactive force fields. In such cases, reactive potentials capable of explicitly modeling bond formation and breaking would be required, which is beyond the scope of the present work. Nevertheless, for the present objective of predicting thermal boundary conductance at room temperature, the classical force field provides an appropriate and sufficient description. We then switch to the NVT ensemble (constant number of atoms, volume, and temperature) and equilibrate for an additional 1 ns. After equilibration, we randomly generate Li^+^ ions at the very thin interfacial gap region between the LCO and electrolyte domains to achieve the desired Li^+^ adsorption coverage at the interface, which is varied between 0 and 2 lithium ions per nm^–2^ as is typical for charge–discharge cycles. Prior work by Aggarwal et al.^18^ uses similar lithium surface coverage values to investigate energy barriers to lithium adsorption in electrolytes with various salt species. Using the Gouy–Chapman model,^66^ this ion surface coverage corresponds to an approximate surface potential of 4.15–4.65 V. The maximum surface potential corresponds to the upper limit of lithium ion adsorption based on kinetic energy barriers at this surface potential.^18^ It is important to note that this surface potential does not correspond to the full-cell potential of a realistic battery, which cannot be determined from our simulations as lithium ions do not enter the cathode structure and affect the cathode potential.

To predict the thermal boundary conductances at different lithium ion coverages on the LCO surfaces, we apply a harmonic restraint of 10 kcal/mol to restrict their movement perpendicular to the interface. This ensures that the placed Li^+^ ions do not enter the LCO structure or the electrolyte domain; however, the equilibrium positions of the Li^+^ ions over time do not change to preserve steady-state conditions. To ensure our choice of harmonic restraint does not significantly impact the thermal boundary conductance, we perform additional simulations where the harmonic restraint is increased up to 50 kcal/mol as shown in Figure S5 of the Supporting Information, where no significant change in the calculated thermal boundary conductance is observed.

For the thermal boundary conductance calculations, we establish walls at either end of the domain with 6 Å thicknesses by freezing only the atoms within these regions. We establish a hot and cold bath adjacent to these walls with 6 Å thicknesses and add/remove energy from the hot/cold baths, respectively, which establishes a linear (and steady-state) temperature gradient across the domain. We bin the atoms in the system into 100 equally spaced bins along the direction of the applied heat flux and average the atomic temperature along the orthogonal directions, yielding a one-dimensional temperature gradient. Note, after activating the heat baths, we allow the steady-state temperature gradient to develop over 2 ns before collecting temperature gradient data over an additional 4 ns. Through this approach, the thermal boundary conductances can be calculated as

$$h_{K}=\frac{Q}{\Delta T}$$ 2

where *h*_K_ is the thermal boundary conductance, *Q* is the applied heat flux, and Δ*T* is the temperature drop across the interface. The use of hot and cold baths at the ends of the LCO and polymer electrolyte domains is not intended to replicate realistic operating temperature profiles, but to enable extraction of the intrinsic thermal boundary conductance. Additional simulations with reversed thermostat placement (hot bath on the polymer electrolyte side, cold bath on the LCO side) yield similar TBC values, confirming that our results represent the intrinsic property of the interface rather than an artifact of boundary conditions (Figure S12). To ensure that our TBC values are independent of the size of the LCO structure, as well as our choice of production time (2 ns), we performed additional simulations where we increase the LCO structure length by up to 10× and our run time by up to 3× before extracting data, as shown in Figures S6 and S7, respectively. Our calculated TBC values are not affected by the length of the LCO domain or the production time, signifying a proper choice of parameters for our simulations.

Similarly to the temperature gradient, we also spatially bin the mass density of the system along the *y*-direction in 100 equally spaced bins in order to observe the structural variation of our system near the interface. For all simulations, we use a time step of 0.5 fs. We perform 5 independent simulations at each Li^+^ surface coverage condition to obtain averages of *h*_K_ values reported in this work. We also note that the values we obtain for the thermal conductivity of the liquid electrolyte systems (κ_LiTFSI_ ∼ 0.11 W m^–1^ K^–1^ and κ_LiPF_6__ ∼ 0.12 W m^–1^ K^–1^) agree well with experimentally reported values as shown in Figures S1 and S2 of the Supporting Information. Our calculations of the thermal conductivity of our model LCO domain, κ_LCO_ ∼ 97 W m^–1^ K^–1^ (as carried out under the Green–Kubo formalism; see Figure S2), agrees with prior simulation results.

In order to gain a deeper understanding of the vibrational dynamics that affect interfacial thermal transport across the electrode/electrolyte interface, we calculate the vibrational density of states (DOS) for the adsorbed Li^+^ ions, and the electrode and electrolyte near the interface. When calculating the DOS of the electrode and electrolyte, we consider atoms within 1 nm of the electrode/electrolyte interface. The DOS is proportional to the Fourier transform ( F ) of the velocity autocorrelation function (VACF). We extract the atomic velocities every 10 timesteps for a total of 2 ns, and calculate the DOS via the Welch method of power spectral density, given by,^67^

$$D(\omega )=\frac{1}{2}mF(\mathrm{VACF})\frac{1}{k_{b}T}\rho$$ 3

where *m* is the atomic mass, *k*_b_ is the Boltzmann constant, *T* is the temperature, and ρ is the atomic density.

To gain insight into the relative modal contributions to the interfacial heat transfer, we calculate the spectral contributions of each mode to the interfacial heat flux. In order to calculate these relative contributions, we calculate the heat flux between two groups of atoms, given by,^53^

$$q_{A\to B}(\omega )=\frac{2}{AM\Delta t_{s}}\sum_{j\in B}\sum_{i\in A}\langle {\tilde{F}}_{i}^{B}(\omega )˙{\tilde{v}}_{i}(\omega {)}^{*}\rangle$$ 4

where *ṽ*_*i*_ and *F̃* are the Fourier transforms of the velocity and force vectors, *A* is the surface area, *M* is the number of samples, and Δ*t*_s_ is the sampling interval.

## Supplementary Material



## Notes

Detailed descriptions of the molecular dynamics equilibration procedure, thermal conductivity calculations, the thermal resistor model, time- and size-convergence analyses, and the parameters used for the interatomic potentials (PDF)
