# Supplementary material for: Conducting Salts Govern Thermal Boundary Conductance across Solid Electrode/Organic Liquid Electrolyte Interfaces in Lithium-Ion Batteries
Source: ACS Nano. 2025 Dec 2;19(49):41595–604. doi: 10.1021/acsnano.5c13221 (PMC12713785; doi:10.1021/acsnano.5c13221)
Supplement: Supplementary file 1 [file nn5c13221_si_001.pdf]

Supporting Information: Conducting Salts Govern Thermal Boundary Conductance Across Solid Electrode/Organic Liquid Electrolyte Interfaces in Lithium-ion Batteries

C. Jaymes Dionne,[†] Patrick E. Hopkins,[‡,¶,§] Arijit Bose,[‖] and Ashutosh Giri[*,†]

†*Department of Mechanical, Industrial and Systems Engineering, University of Rhode Island, Kingston, RI 02881, USA*

‡*Department of Mechanical and Aerospace Engineering, University of Virginia, Charlottesville, Virginia 22904, USA*

¶*Department of Materials Science and Engineering, University of Virginia, Charlottesville, Virginia 22904, USA*

§*Department of Physics, University of Virginia, Charlottesville, Virginia 22904, USA*

‖*Department of Chemical Engineering, University of Rhode Island, Kingston, RI 02881, USA*

E-mail: ashgiri@uri.edu

Supplementary Note 1: Equilibration Procedure

Once the structures are generated, we perform an energy minimization of the structure at 0 K using the conjugate gradient (CG) algorithm with stopping tolerances of 1×10^{-4} and 1×10^{-6} for the energy and force, respectively. After minimization, we initialize the system at 300 K. We allow the system to relax for 2 ns under the NPT ensemble (constant number of atoms, pressure, and temperature held constant) for the given temperature of 300 K and ambient pressure, and confirm the total volume of the system and total energy have equilibrated, as shown in Supplementary Figure 9. Once the system volume has equilibrated, we perform additional relaxation under the NVT ensemble (constant number of atoms, volume, and temperature held constant) at 300 K for 1 ns to ensure the system pressure and total energy is equilibrated, as shown in Supplementary Figure 10. Finally, to ensure the system follows Newtonian dynamics, we simulate for an additional 1 ns under the NVE ensemble (constant number of atoms, volume, and total energy held constant) as shown in Supplementary Figure 11. After proper equilibration is achieved, we establish our heat baths under the NEMD framework and calculate the thermal boundary conductance as outlined in the Methods section of the main text.

Supplementary Note 2: Thermal Conductivity Calculations

To calculate the thermal conductivity of our liquid electrolyte, we extracted the temperature gradient from our simulations following the procedure laid out in the main text. By using the temperature gradient in the liquid electrolyte and our applied heat flux, the thermal conductivity can be computed by

$$\kappa = \frac{Q}{\Delta T} \tag{1}$$

where Q is the applied heat flux and ΔT is the temperature gradient. As shown in Supplementary Figure 1, the thermal conductivity of the liquid electrolyte agrees well with experimentally obtained results.

In our NEMD simulations, the LCO domain is too small to resolve a thermal conductivity from the temperature gradient alone. Instead, we perform Green-Kubo (GK) calculations on an equivalently sized LCO single crystal. The thermal conductivity in the GK approach is given by,[S1]

$$\kappa_\alpha = \frac{1}{k_B V T^2} \int_0^\infty \langle S_\alpha(t) S_\alpha(0) \rangle dt, \tag{2}$$

where t is the time, T and V are the temperature and volume of the system, and $\langle S_\alpha(t) S_\alpha(0) \rangle$ is the αth component of the heat current autocorrelation function (HCACF). The heat current vector is given by,[S1]

$$S_\alpha = \frac{d}{dt} \sum r_i E_i, \tag{3}$$

where r_i and E_i are the position vector and energy (kinetic and potential) of the ith particle in a system, respectively.

Supplementary Table 1: Calculated thermal conductivities of our polymer electrolytes and LCO cathode.

	Calculated κ (W m^{-1} K^{-1})	Reference κ (W m^{-1} K^{-1})
$LiPF_6$	0.11	0.14 - 2.1 [S2, S3]
LiTFSI	0.12	0.14 - 2.1 [S2, S3]
LCO	97.8 ($Li(CoO_2)_2$)	~147 ($Li(CoO_2)$ [S4]

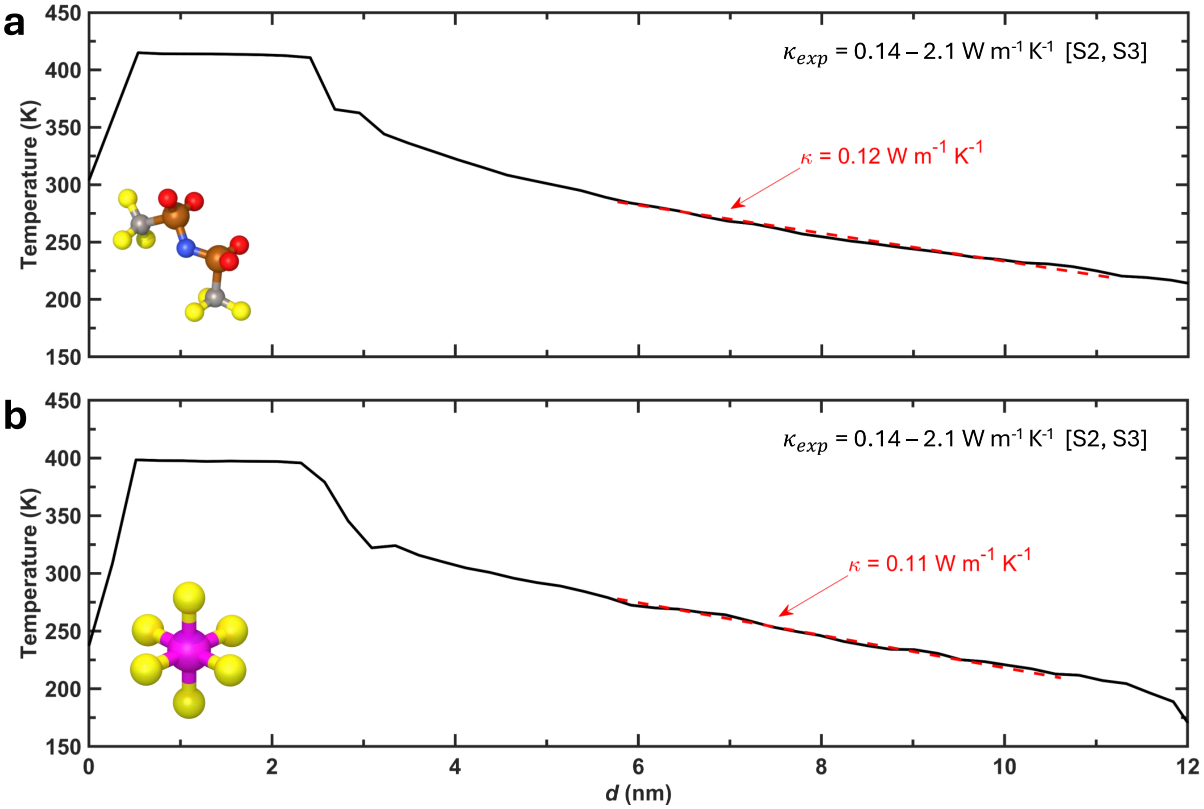

Supplementary Figure 1: Thermal conductivity of our (a) LiTFSI and (b) $LiPF_6$ electrolytes obtained via the NEMD method. The calculated values agree well with experimentally determined values from refs. S2 and S3.

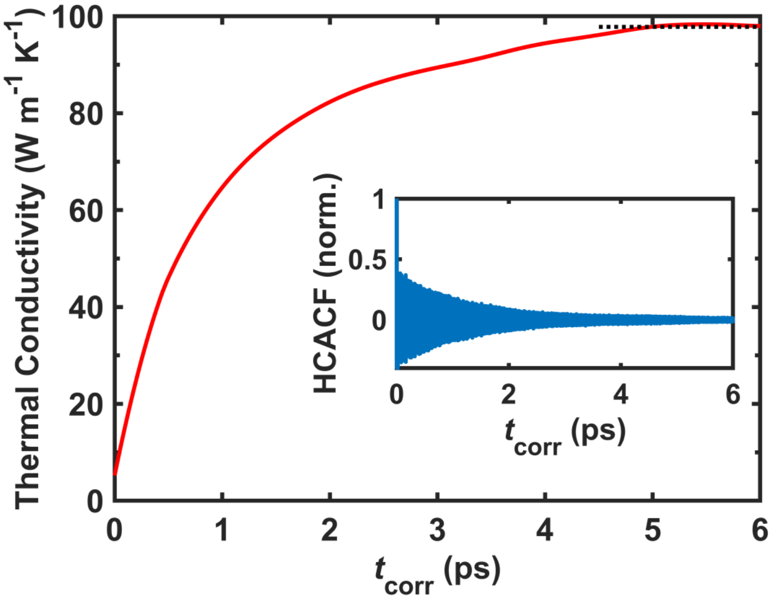

Supplementary Figure 2: Thermal conductivity in the [110] plane of our LCO $(Li(CoO_2)_2$ structure obtained via the GK method. The calculated values agree well with computationally determined values for single crystal $LiCoO_2$ ($\sim$147 W m^{-1} K^{-1}).[S4] Our calculated value is lower due to the delithiated structure, which has been shown to reduce the thermal conductivity of single crystal LCO by up to 70%.[S5]

Supplementary Note 3: Thermal Resistor Model

We implement a thermal resistor model to determine the relative contribution of the interface resistance to the overall resistance within the cathode. The total thermal resistance of the system is given by,

$$R_{total} = R_{electrode} + R_{interface} + R_{electrolyte} \tag{4}$$

where $R_{electrode} = l_{electrode}/\kappa_{electrode}$, $R_{interface} = 1/h_k$, and $R_{electrolyte} = l_{electrolyte}/\kappa_{electrolyte}$. As the thermal conductivity of LCO is large compared to that of the electrolyte, we neglect the contribution to total thermal resistance from the electrode. We use a range of lengths for the liquid electrolyte corresponding to a pore size variation of ~10 nm to >1 μm[S6,S7] to determine the resistance contributed by the liquid electrolyte. The contribution to the total resistance by the interface can then be calculated as,

$$R_{cont.} = \frac{R_{total}}{R_{interface}} \tag{5}$$

Supplementary Note 4: Density of State Overlap

In order to determine the extent of vibrational overlap between the LCO and liquid electrolyte, we calculate the overlap percentage by integrating the vibrational density of states shared by both the LCO and electrolyte. This overlap area is shown in Supplementary Figure 13 as the shaded blue region. We only consider modes ≤ 20 THz, as these are the dominant modes for interfacial heat transport determined from our spectral calculations (Fig. 4 of the main text). To modify the vibrational spectrum of LCO, we create a fictitiously heavy LCO structure by increasing the average atomic mass by 100% and a fictitiously light structure by decreasing the average atomic mass by 50%. As shown in Supplementary Figure 14 for a bare LCO/electrolyte interface, the increase in vibrational overlap resulting from the modification

of the LCO vibrational spectrum yields an increase in the calculated thermal boundary conductance. As lithium ions adsorb onto the LCO surface, this vibrational overlap increases and results in a further increase in the calculated thermal boundary conductance. It is important to note that the extent of vibrational overlap between the LCO and electrolyte does not fully determine interfacial heat transfer, as is evident from the discrepancy between calculated thermal boundary conductances at similar overlap percentages shown in Supplementary Figure 14. Instead, additional effects like liquid structuring of density depletion play significant roles in interfacial heat transfer at solid/liquid interfaces.

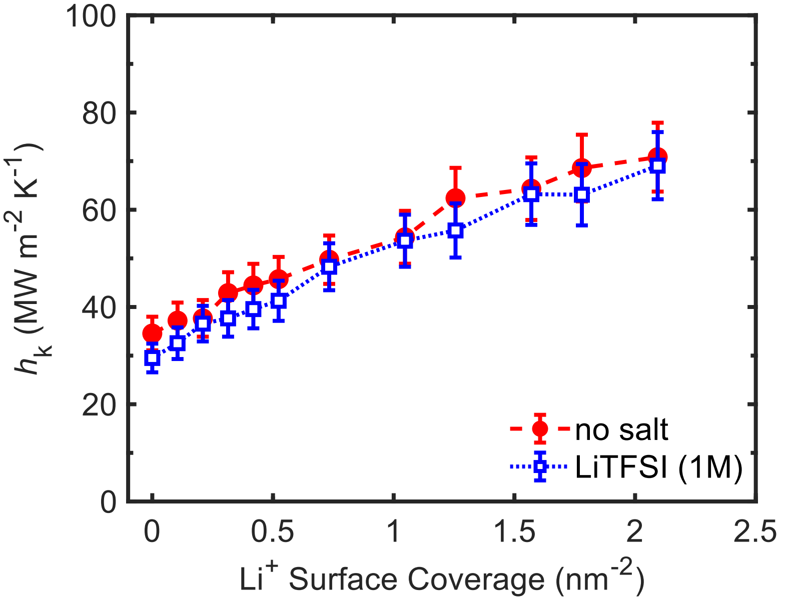

Supplementary Figure 3: Thermal boundary conductance as a funciton of lithium ion surfce coverage for the cases with no salt (solid circles) and for 1M LiTFSI.

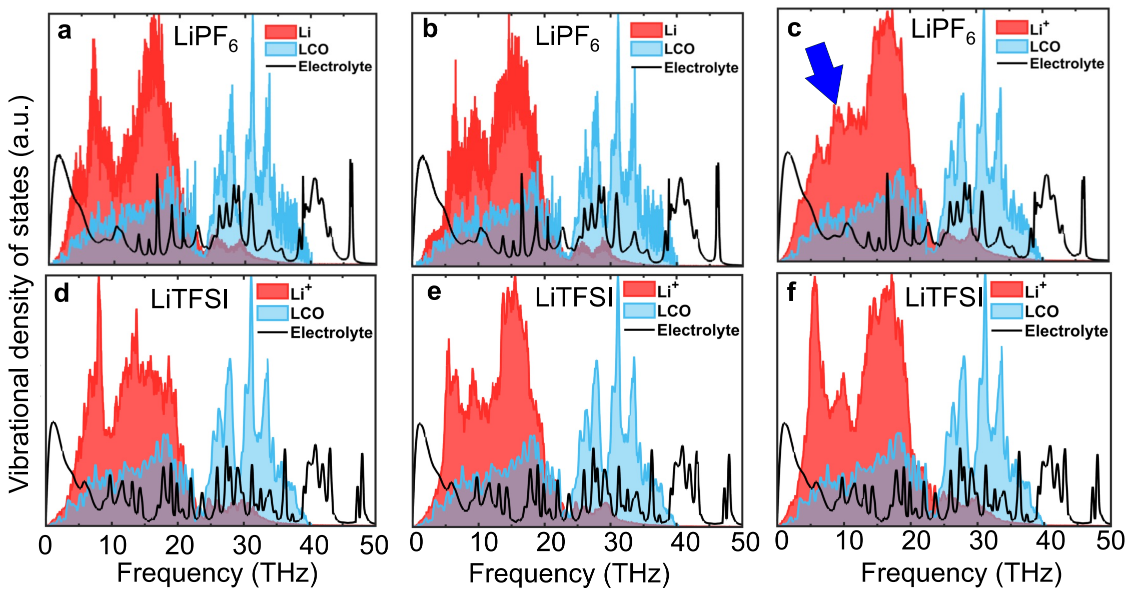

Supplementary Figure 4: Vibrational density of states of the electrolyte, the LCO electrode, and the lithium ions adsorbed at the surface of LCO at varying conditions of lithium adsorption. The top panels (a-c) are for the $LiPF_6$ system and the bottom panels (d-f) are for the LiTFSI system. The lithium coverages are (a,d) 0.5 nm^{-2}, (b,e) 1 nm^{-2}, and (c,f) 2 nm^{-2}.

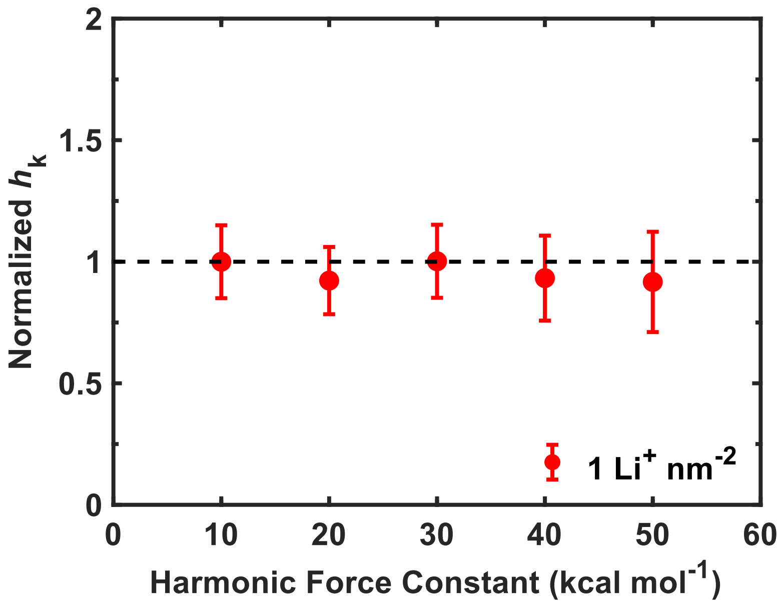

Supplementary Figure 5: Normalized thermal boundary conductance for the 1M LiTFSI system with 1 Li^+ nm^{-2} adsorbed on the electrode surface as a function of the applied harmonic restraint to the adsorbed lithium ions. The values are normalized to the thermal boundary conductance value reported in the main text for the LCO/LiTFSI interface at 1 Li^+ nm^{-2}. Increasing the strength of the harmonic restraint does not have a significant impact on the calculated thermal boundary conductances in our simulations.

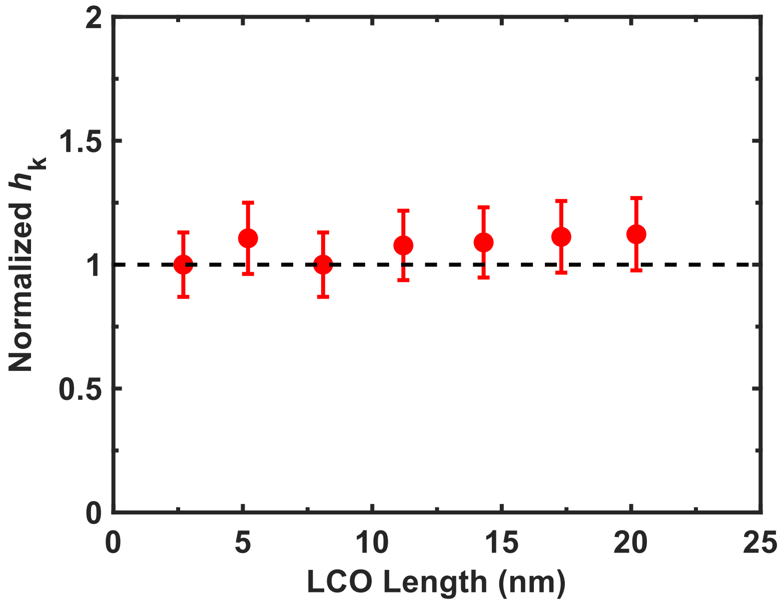

Supplementary Figure 6: Normalized thermal boundary conductance for the 1M LiTFSI system with increasing length of the solid LCO structure. The values are normalized to the thermal boundary conductance value reported in the main text for the LCO/LiTFSI interface at 0 Li^+ nm^{-2}. The calculated thermal boundary conductance does not significantly change, even for a tenfold increase in the length of the LCO structure.

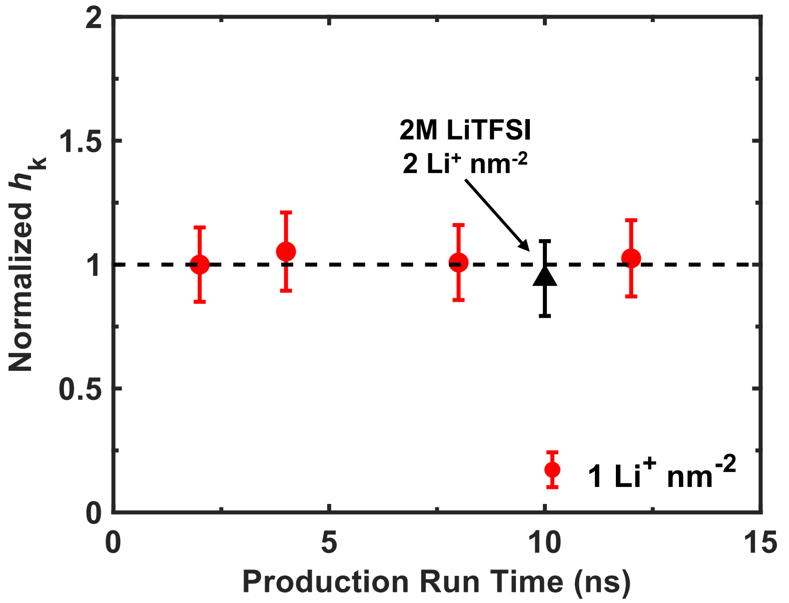

Supplementary Figure 7: Normalized thermal boundary conductance for the 1M LiTFSI system with 1 Li^+ nm^{-2} (red) adsorbed on the electrode surface as a function of the production run time of our NEMD simulations. The values are normalized to the thermal boundary conductance value reported in the main text for the LCO/LiTFSI interface at 1 Li^+ nm^{-2}. The calculated thermal boundary conductance does not significantly change as the production time is increased, signifying that our simulations have reached equilibrium before we begin extracting data. The calculated thermal boundary conductance for the 2M LiTFSI at maximum Li^+ surface coverage (2 Li^+ nm^{-2}) also remains unchanged for longer production run times.

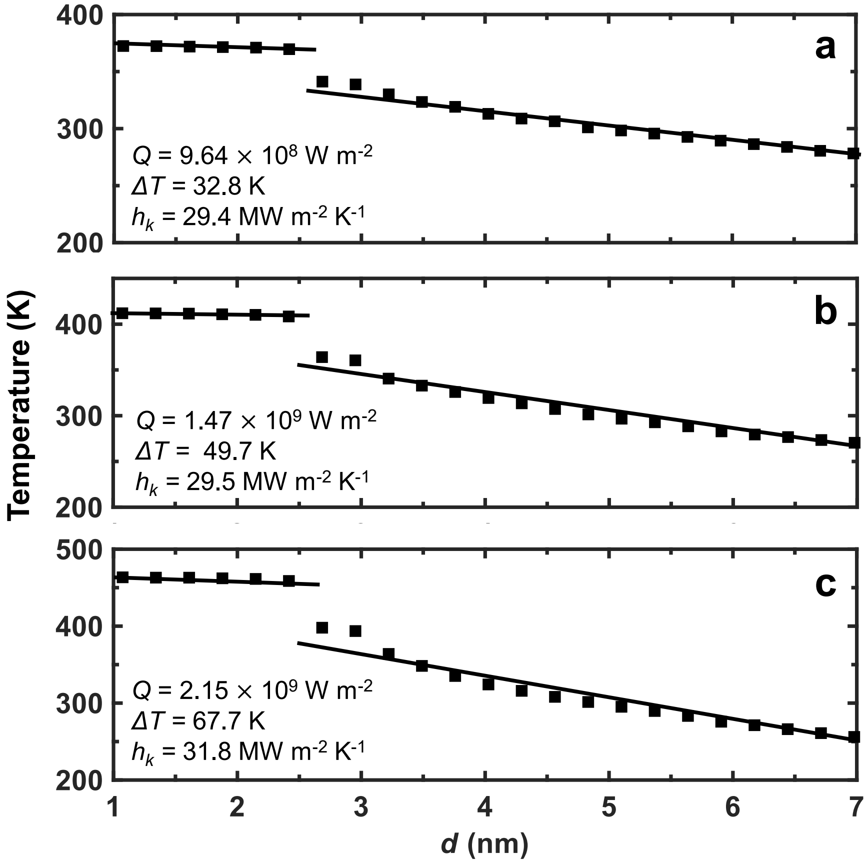

Supplementary Figure 8: Thermal boundary conductances for the 1M LiTFSI system as a function of applied heat flux. The calculated thermal boundary conductance does not significantly change over the range of tested heat fluxes, and we use a value of Q=1.47×10^9 W m^{-2} K^{-1} to calculate the thermal boundary conductances in the main text.

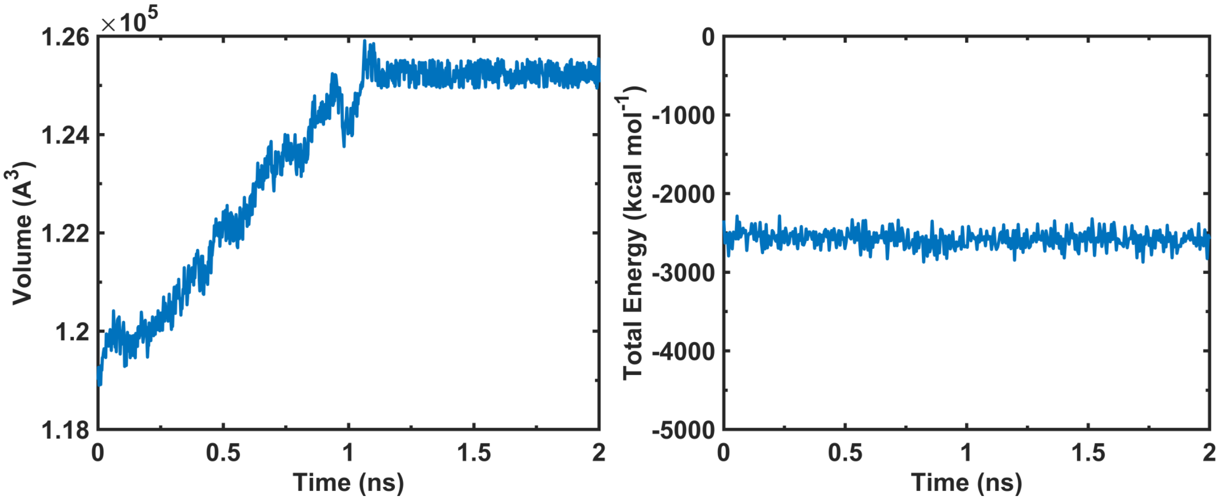

Supplementary Figure 9: (a) Volume and (b) energy traces during our NPT equilibration after energy minimization at 0 K and application of a random velocity distribution at 300 K.

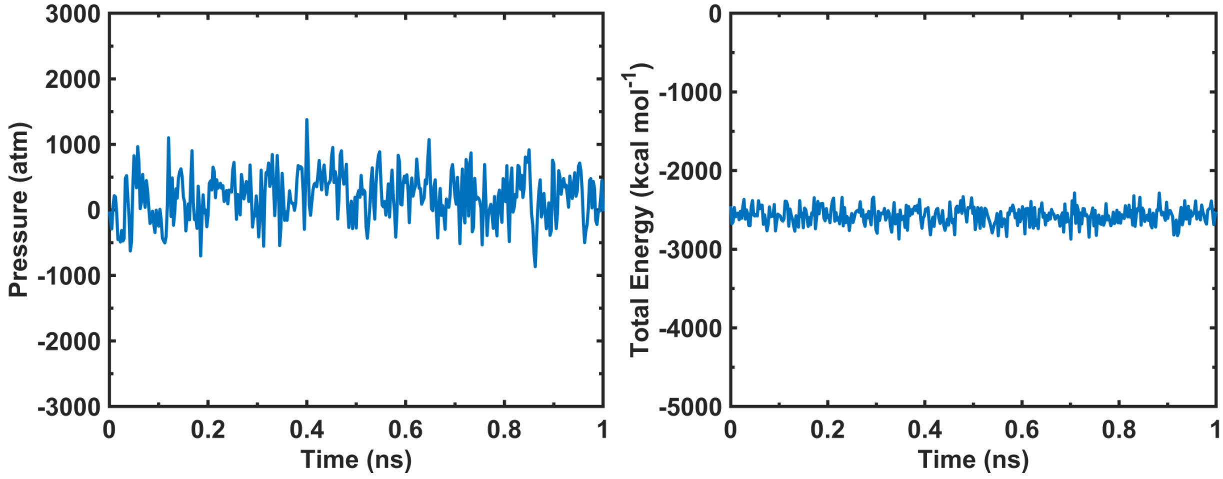

Supplementary Figure 10: (a) Pressure and (b) energy traces during our NVT equilibration.

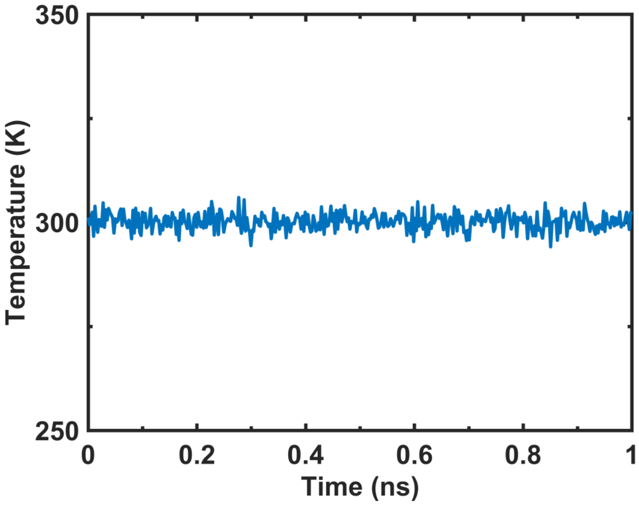

Supplementary Figure 11: Temperature trace during our NVE equilibration.

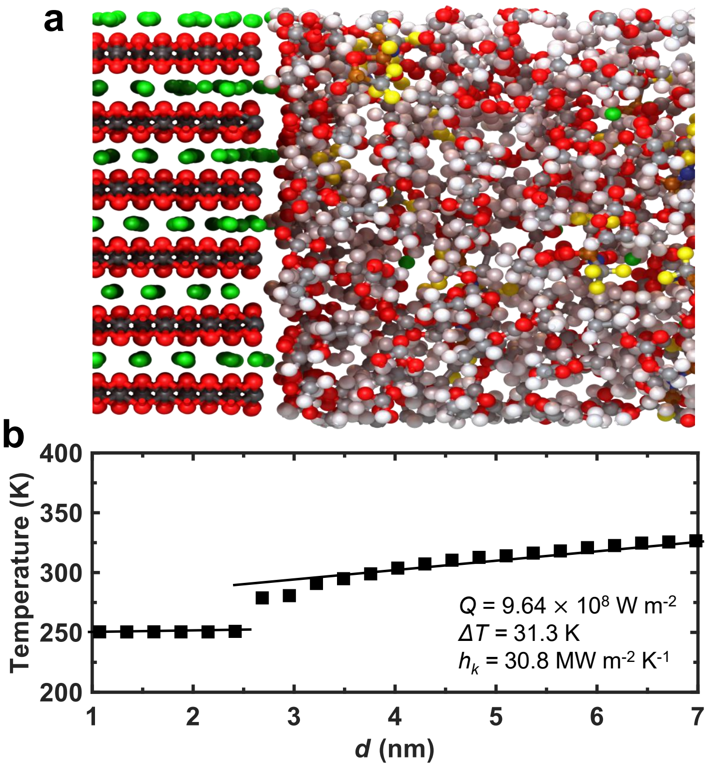

Supplementary Figure 12: Calculated thermal boundary conductance with hot bath placed within electrolyte and cold bath placed within electrode. The calculated thermal boundary conductance agrees well with results presented in the main text, indicating this value is an intrinsic property of the interface.

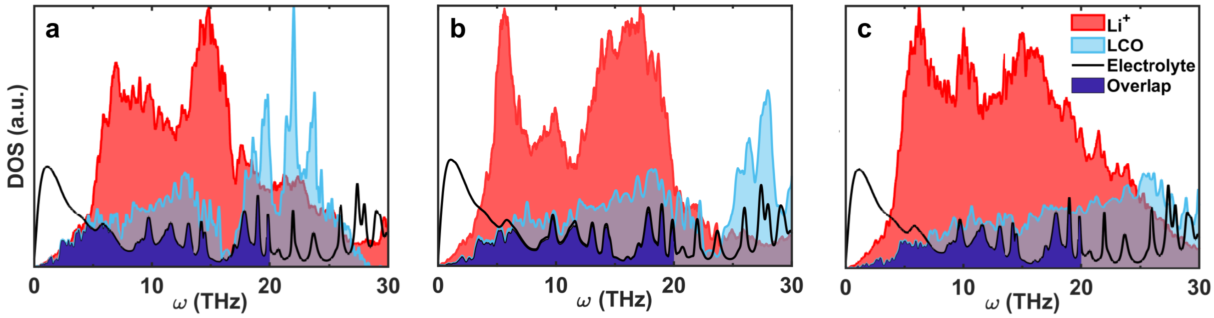

Supplementary Figure 13: Density of states overlap between LCO and electrolyte for (a) a fictitiously heavy LCO with a narrower frequency spectrum, (b) regular LCO, and (c) a fictitiously light LCO with a broader frequency spectrum. The overlap in DOS between the LCO and electrolyte increases primarily in the ≤10 THz regime, resulting in increased interfacial heat transfer across the LCO/electrolyte inferface. The DOS overlap is only calculated in the ≤20 THz regime.

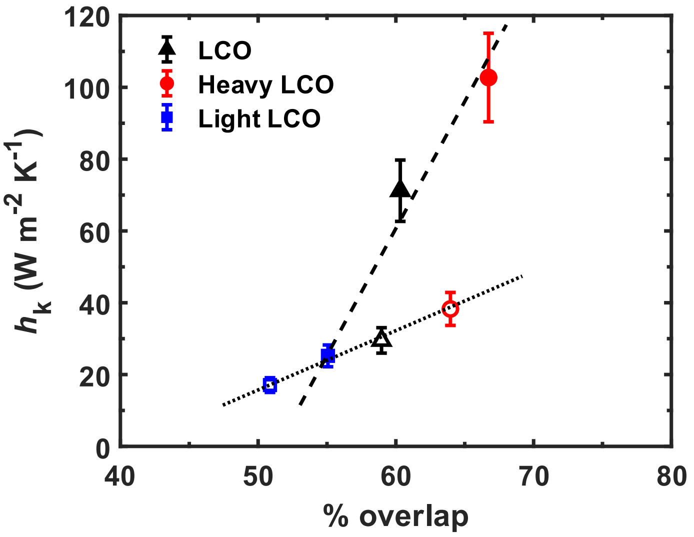

Supplementary Figure 14: Calculated thermal boundary conductance as a function of the DOS overlap between the regular, fictitiously heavy, and fictitiously light LCO and electrolyte in the ≤20 THz range. Hollow and filled markers correspond to no lithium adsorption and 2 nm^{-2} lithium adsorption, respectively. An increase in the overlap of DOS increases the interfacial heat transfer across the LCO/electrolyte interface, with lithium adsorption resulting in an increase in DOS overlap and concomitant increase in thermal boundary conductance.

$LiPF_6$ Forcefield Parameters

ATOMS
Name, Type, Mass(u), Charge(e), Potential type, σ (Å), ε (kJ/mol)
Li cation
Li Li 6.941 +1.00 lj 2.126 0.07648
hexafluorophosphate JCSPerkin2 (1999) 2365
P P 30.974 1.34 lj 3.74 0.83680
FP F 18.998 -0.39 lj 3.12 0.25520

BONDS
Atom 1 type, Atom 2 type, Bond potential type, Equilibrium distance (Å), Force Constant (kJ/mol/Å^2)
hexafluorophosphate JCSPerkin2(1999)2365
P F harm 1.606 3100.0

ANGLES
Atom 1 type, Atom 2 type, Atom 3 type, Angle potential type, Equilibrium angle (°), Force constant (kJ/mol/rad^2)
hexafluorophosphate JCSPerkin2(1999)2365
F P F harm 90.0 1165.0

LiTFSI Forcefield Parameters

ATOMS
Name, Type, Mass(u), Charge(e), Potential type, σ (Å), ε (kJ/mol)
lithium cation Aqvist
Li Li 6.941 +1.00 lj 2.126 0.07648
bistriflamide JPCB 108 (2004) 16893, PCCP 43 (2017) 29617
CBT CF 12.011 0.35 lj 3.50 0.27614
SBT SB 32.066 1.02 lj 3.55 1.04600
NBT NB 14.000 -0.66 lj 3.25 0.71128
OBT OB 15.999 -0.53 lj 3.15 0.83736
F1 FB 18.998 -0.16 lj 3.118 0.25540

BONDS
Atom 1 type, Atom 2 type, Bond potential type, Equilibrium distance (Å), Force Constant (kJ/mol/Å^2)
triflate and bistriflamide JPCB108(2004)16893
FB CF harm 1.323 3698.0
CF SB harm 1.818 1950.0
SB OB harm 1.437 5331.0
NB SB harm 1.570 3137.0

ANGLES
Atom 1 type, Atom 2 type, Atom 3 type, Angle potential type, Equilibrium angle (°), Force constant (kJ/mol/rad^2)
triflate and bistriflamide JPCB108(2004)16893
FB CF FB harm 107.1 781.0
FB CF SB harm 111.7 694.0
OB SB OB harm 118.5 969.0
CF SB OB harm 102.6 870.0
NB SB OB harm 113.6 789.0
NB SB CF harm 103.5 764.0
SB NB SB harm 125.6 671.0

DIHEDRALS
Atom 1 type, Atom 2 type, Atom 3 type, Atom 4 type, Bond potential type, V1 (kJ/mol), V2 (kJ/mol), V3 (kJ/mol), V4 (kJ/mol)
triflate and bistriflamide JPCB 108 (2004) 16893
OB SB CF FB opls 0.0000 0.0000 1.4510 0.0000
NB SB CF FB opls 0.0000 0.0000 1.3220 0.0000
OB SB NB SB opls 0.0000 0.0000 -0.0150 0.0000
SB NB SB CF opls 32.7730 -10.4200 -3.1950 0.0000

<u>EC Forcefield Parameters</u>

ATOMS
Name, Type, Mass(u), Charge(e), Potential type, σ (Å), ε (kJ/mol)
carbonates OPLS
C_2 C_2 12.011 0.784 lj 3.75 0.43932
O_2 O_2 15.999 -0.508 lj 2.96 0.87864
OS OS 15.999 -0.246 lj 3.00 0.71128
CM CM 12.011 -0.068 lj 3.55 0.31798
HCM HC 1.008 0.088 lj 2.42 0.12552

BONDS
Atom 1 type, Atom 2 type, Bond potential type, Equilibrium distance (Å), Force Constant (kJ/mol/Å^2)
carbonates
C_2 O_2 harm 1.229 4769.8
C_2 OS harm 1.370 1790.8
CM OS harm 1.370 3765.6
CM CM harm 1.340 4594.0
CM HC cons 1.080 2845.1

ANGLES
Atom 1 type, Atom 2 type, Atom 3 type, Angle potential type, Equilibrium angle (°), Force constant (kJ/mol/rad^2)
carbonates Kosmus (angles) OPLS (force consants)
O_2 C_2 OS harm 125.6 694.5
OS C_2 OS harm 108.8 694.5
C_2 OS CM harm 107.0 585.8
OS CM CM harm 108.6 585.8
OS CM HC harm 109.5 292.9
CM CM HC harm 125.7 292.9
HC CM HC harm 107.8 276.144

DIHEDRALS
Atom 1 type, Atom 2 type, Atom 3 type, Atom 4 type, Bond potential type, V1 (kJ/mol), V2 (kJ/mol), V3 (kJ/mol), V4 (kJ/mol)
carbonates
O_2 C_2 OS CM opls 0.0000 21.4388 0.0000 0.0000
CM OS C_2 OS opls -1.5690 -5.6819 0.0167 0.0000
C_2 OS CM CM opls -14.6440 12.5520 0.0000 0.0000
C_2 OS CM HC opls 0.0000 0.0000 3.1798 0.0000
OS CM CM OS opls 0.0000 58.5760 0.0000 0.0000
OS CM CM HC opls 0.0000 58.5760 0.0000 0.0000
HC CM CM HC opls 0.0000 58.5760 0.0000 0.0000

EMC Forcefield Parameters

ATOMS
C00 C00 12.011 -0.2472 lj 3.50 0.27614
H01 H01 1.008 0.1034 lj 2.50 0.12552
H02 H02 1.008 0.1034 lj 2.50 0.12552
H03 H03 1.008 0.1034 lj 2.50 0.12552
C04 C04 12.011 0.0296 lj 3.50 0.27614
H05 H05 1.008 0.1062 lj 2.50 0.12552
H06 H06 1.008 0.1062 lj 2.50 0.12552
O07 O07 15.999 -0.3389 lj 2.90 0.58576
C08 C08 12.011 0.5783 lj 3.55 0.29288
O09 O09 15.999 -0.5053 lj 2.96 0.87864
O0A O0A 15.999 -0.3307 lj 2.90 0.58576
C0B C0B 12.011 -0.0326 lj 3.50 0.27614
H0C H0C 1.008 0.1080 lj 2.50 0.12552
H0D H0D 1.008 0.1080 lj 2.50 0.12552
H0E H0E 1.008 0.1082 lj 2.50 0.12552

BONDS
H01 C00 cons 1.090 2845.1
H02 C00 cons 1.090 2845.1
H03 C00 cons 1.090 2845.1
C04 C00 harm 1.529 2242.6
H05 C04 cons 1.090 2845.1
H06 C04 cons 1.090 2845.1
O07 C04 harm 1.410 2677.8
C08 O07 harm 1.327 1790.8
O09 C08 harm 1.229 4769.8
O0A C08 harm 1.327 1790.8
C0B O0A harm 1.410 2677.8
H0C C0B cons 1.090 2845.1
H0D C0B cons 1.090 2845.1
H0E C0B cons 1.090 2845.1

ANGLES
H01 C00 H02 harm 107.8 276.1
H01 C00 H03 harm 107.8 276.1
H01 C00 C04 harm 110.7 313.8
C00 C04 H05 harm 110.7 313.8
C00 C04 H06 harm 110.7 313.8
C00 C04 O07 harm 109.5 418.4
C04 O07 C08 harm 116.9 694.5
O07 C08 O09 harm 123.4 694.5
O07 C08 O0A harm 118.2 584.9
C08 O0A C0B harm 116.9 694.5
O0A C0B H0C harm 109.5 292.9
O0A C0B H0D harm 109.5 292.9
O0A C0B H0E harm 109.5 292.9
H0C C0B H0D harm 107.8 276.1
H03 C00 C04 harm 110.7 313.8
H02 C00 C04 harm 110.7 313.8
H0C C0B H0E harm 107.8 276.1
H0D C0B H0E harm 107.8 276.1
H06 C04 O07 harm 109.5 292.9
H05 C04 H06 harm 107.8 276.1
H02 C00 H03 harm 107.8 276.1
H05 C04 O07 harm 109.5 292.9
O09 C08 O0A harm 123.4 694.5

DIHEDRALS
C08 O07 C04 C00 opls 5.2010 0.5270 3.5310 0.0000

C08 O07 C04 H06 opls 1.2430 0.0000 1.6570 0.0000
C08 O07 C04 H05 opls 1.2430 0.0000 1.6570 0.0000
C0B O0A C08 O09 opls 0.0000 21.4390 0.0000 0.0000
C0B O0A C08 O07 opls 9.7680 21.4390 0.0000 0.0000
H06 C04 C00 H01 opls 1.8830 0.0000 2.5100 0.0000
H05 C04 C00 H01 opls 1.8830 0.0000 2.5100 0.0000
H05 C04 C00 H03 opls 1.8830 0.0000 2.5100 0.0000
H06 C04 C00 H03 opls 1.8830 0.0000 2.5100 0.0000
H06 C04 C00 H02 opls 1.8830 0.0000 2.5100 0.0000
H05 C04 C00 H02 opls 1.8830 0.0000 2.5100 0.0000
H0C C0B O0A C08 opls 1.2430 0.0000 1.6570 0.0000
H0D C0B O0A C08 opls 1.2430 0.0000 1.6570 0.0000
H0E C0B O0A C08 opls 1.2430 0.0000 1.6570 0.0000
O09 C08 O07 C04 opls 0.0000 21.4390 0.0000 0.0000
O0A C08 O07 C04 opls 9.7680 21.4390 0.0000 0.0000
O07 C04 C00 H01 opls 2.9370 0.0000 3.9160 0.0000
O07 C04 C00 H02 opls 2.9370 0.0000 3.9160 0.0000
O07 C04 C00 H03 opls 2.9370 0.0000 3.9160 0.0000

IMPROPERS
O07 O09 C08 O0A opls 0.0000 43.9320 0.0000 0.0000

Supplementary References

(S1) Wang, Z.; Safarkhani, S.; Lin, G.; Ruan, X. Uncertainty quantification of thermal conductivities from equilibrium molecular dynamics simulations. *International Journal of Heat and Mass Transfer* **2017**, *112*, 267 – 278.

(S2) Sederholm, J. G.; Ahmari, A.; Yeon, S.; Zhou, J.; Griebler, J. J.; Cahill, D. G.; Braun, P. V. Thermal Conductivity of Battery Electrolytes and Binary Electrolyte Mixtures. *ECS Meeting Abstracts* **2025**, *MA2025-01*, 832, Publisher: The Electrochemical Society, Inc.

(S3) Werner, D.; Loges, A.; Becker, D. J.; Wetzel, T. Thermal conductivity of Li-ion batteries and their electrode configurations – A novel combination of modelling and experimental approach. *Journal of Power Sources* **2017**, *364*, 72–83.

(S4) He, J.; Zhang, L.; Liu, L. Thermal transport in monocrystalline and polycrystalline lithium cobalt oxide. *Physical Chemistry Chemical Physics* **2019**, *21*, 12192–12200, Publisher: The Royal Society of Chemistry.

(S5) Feng, T.; O'Hara, A.; Pantelides, S. T. Quantum prediction of ultra-low thermal conductivity in lithium intercalation materials. *Nano Energy* **2020**, *75*, 104916.

(S6) Das, P. R.; Komsiyska, L.; Osters, O.; Wittstock, G. PEDOT: PSS as a Functional Binder for Cathodes in Lithium Ion Batteries. *Journal of The Electrochemical Society* **2015**, *162*, A674.

(S7) Beuse, T.; Fingerle, M.; Wagner, C.; Winter, M.; Börner, M. Comprehensive Insights into the Porosity of Lithium-Ion Battery Electrodes: A Comparative Study on Positive Electrodes Based on LiNi0.6Mn0.2Co0.2O2 (NMC622). *Batteries* **2021**, *7*.
